# Etiologies des accidents vasculaires cérébraux ischémiques chez les jeunes: apport de l’interniste

**DOI:** 10.11604/pamj.2018.30.114.13879

**Published:** 2018-06-12

**Authors:** Abire Allaoui, Khadija Echchilali, Mina Moudatir, Fatim Zohra Alaoui, Hassan Elkabli

**Affiliations:** 1 Service de Médecine Interne, CHU Ibn Rochd, Casablanca, Faculté de Médecine Privée Casablanca, Université Hassan II, Maroc

**Keywords:** Ischémie cérebrale, sujets jeunes, étiologie, Brain ischemia, young people, cause

## Abstract

Les ischémies cérébrales chez les jeunes sont différentes par leurs étiologies et leur pronostic. Le profil étiologique en médecine interne est particulier vu le niveau tertiaire de recrutement. L'objet de notre travail est de faire connaître certaines étiologies particulières de cette pathologie chez le sujet jeune. Etude rétrospective des dossiers de jeunes patients au service de médecine interne à Casablanca, sur une période de 2000-2014. Tous les patients ont bénéficié d'une imagerie cérébrale avec des séquences angiographiques, précisant la nature et la topographie de l'AVCI. Un bilan étiologique dicté par la clinique a été mené. Vingt et cinq patients ont été colligés, avec un sex-ratio à 0,73 et un âge moyen de 36±7. Le tabagisme était présent dans 32% des cas, le diabète et l'hypertension artérielle ont été notés dans 8% des cas. Les épisodes d'amnésie et de migraine ont été notés dans 24%. La prise d'oestroprogestatifs et l'antécédent de fausses couches ont été notés dans 12%. Les étiologies étaient dominées par: lupus systémique aigu (32%) associé à un syndrome des antiphospholipides (80%), maladie de Behcet (16%), maladie de Takayasu (12%). En plus de l'antiagrégation, 76% de nos patients ont eu un traitement aux corticoïdes et immuno-suppresseurs. La médecine interne participe à la prise en charge des AVCI, notamment dans le volet étiologique. Les étiologies des ischémies cérébrales du sujet jeune sont multiples, la recherche doit être rigoureuse afin d'identifier des causes particulières, évaluer le risque de récidive et préciser la stratégie thérapeutique.

## Abstract (en)

In young people brain ischemias vary according to their cause and prognosis. In internal medicine they have a specific cause, considering the tertiary source of recruitment. Our study aimed to provide informations about some specific causes of this disease in young subject. We conducted a retrospective study by reviewing the medical records of young patients hospitalized at the Department of Internal Medicine in Casablanca over the period 2000-2014. All patients underwent CT scan angiography and/or magnetic resonance angiography that determined the nature and the topography of the ischemic stroke. Clinical examination was followed by appropriate investigations to determine the cause of brain ischemia. Data were collected from twenty-five patients, with a sex ratio of 0.73 and an average age of 36±7. Smoking was reported in 32% of cases, diabetes and high blood pressure were found in 8% of cases. Amnesia and migraine episodes were found in 24% of cases. Combination estrogen-progestin and a history of miscarriage were reportedin 12% of cases. Ischemic strokes were mainly caused by acute systemic lupus (32%) associated with antiphospholipid syndrome (80%), Behcet’s disease (16%), Takayasu’s disease (12%). In addition to anti-aggregation treatment, 76% of patients underwent corticosteroid and immunosuppressive therapies. Internists are involved in the treatment of patients with ischemic stroke, in particular to determine its cause. The causes of brain ischemias in young people are multiple. Research has to be rigorous in order to identify specific causes, to evaluate the risk of recidivism and to establish a therapeutic approach.

## Introduction

Les accidents vasculaires cérébraux ischémiques (AVCI) constituent un problème de santé majeur, touchant plus de 15 millions de patients chaque année dans le monde, responsable du décès d'un tiers d'entre eux et de séquelles invalidantes chez 2/3 des survivants [1]. Considérés depuis longtemps comme l'apanage des sujets âges, les nou-velles données suggèrent que cette pathologie est bien présente chez les sujets jeunes et est de plus en plus fréquente chez la population de moins 50 ans [2,3]. En plus des causes classiques des AVCI qu'ils partagent avec les sujets âgés, les sujets jeunes présentent d'autres étiologies avec une prédominance des « autres causes déterminées » et des « AVC cryptogéniques » [4]. Il est important de chercher ces causes qu'on pourrait prévenir ou traiter précocement et ainsi diminuer les séquelles fonctionnelles voir la mortalité dans cette population. Un domaine comme la médecine interne regorge de pathologies qui pourraient prédisposer aux AVCI et en font une source de recrutement de ce type de patients. Dans un pays comme le Maroc où les cardiopathies rhumatismales sévissent encore très fortement, et constituent l'une des premières causes d'AVCI chez les jeunes, une revue des patients atteints d'AVCI ayant été recrutés par un service de médecine interne nous a semblé nécessaire afin de faire la lumière sur des étiologies beaucoup plus rares mais non exceptionnelles dont la connaissance pourraient améliorer grandement le pronostic chez ces patients jeunes. Cette étude a comme objectif d'établir un profil étiologique des AVCI chez les jeunes et leurs différentes caractéristiques.

## Méthodes

Pour mener cette étude, un recueil rétrospectif des données à partir des dossiers médicaux a été effectué sur une période allant de 2000-2014. Les critères d'inclusion des patients étaient la présentation par un AVCI, ayant amené le patient à transiter par un service de médecine interne au CHU de Casablanca et ayant un âge inférieur à 45 ans. L'AVCI était défini par la survenue brutale d'un déficit neurologique focal, dont l'origine ischémique a été confirmée par l'imagerie cérébrale. Le recueil des données s'est fait à l'aide d'une fiche qui évaluait l'état civil des patients (âge, sexe), les facteurs de risque vasculaire (hypertension artérielle, hypercholestérolémie, arythmie par fibrillation auriculaire, diabète, tabagisme, antécédents vasculaires cérébraux ou d'autres atteintes vasculaires), les habitudes toxiques, les prises médica-menteuses, la contraception orale, les antécédents obstétricaux ainsi que la présence d'antécédents migraineux. Des épisodes d'aphtose buccale ou génitale, une atteinte oculaire, articulaire ou cutanée ont été aussi notés. La présentation clinique de l'AVCI, le bilan réalisé (électrocardiogramme, scanner cérébral, exploration ultrasonore cervicale, échocardiographie cardiaque transthoracique ou transoesophagienne, holter-électrocardiogramme, IRM encéphalique, bilan de thrombophilie, bilan autoimmune), la prise en charge thérapeutique et le devenir des patients, ont été aussi relevés. Tous les patients ont bénéficié d'une imagerie cérébrale (TDM et/ou IRM) avec des séquences angiographiques, précisant la nature et la topographie de l'AVCI. D'autres examens à visée étiologique dictés par le contexte clinique ont été éffectués : bilan de thrombophilie (antiphospholipides, déficit en protéines de la coagulation. . .), bilan cardiovasculaire (ECG, voire holter ECG, échocardiodoppler, écho-doppler carotidien), bilan autoimmun. Les sous types d'AVCI ont été classés selon la classification TOAST « trial of org 10172 acute stroke treatment » [5]. Le score NIHSS n'a pas été noté sur les dossiers.

## Résultats

Vingt et cinq patients ont été colligés, avec un sex-ratio à 0,73 et un âge moyen de 36±7. Plusieurs facteurs favorisants ont été trouvés chez nos malades. Le tabagisme était présent dans 32% des cas. Le diabète type 2 et l'hypertension artérielle ont été notés chacun dans 8% des cas. Le terrain migraineux et des épisodes d'amnésie précédant l'événement vasculaire ont été relevés dans 24%. La prise d'oestro-progestatifs et l'antécédent de fausses couches à répétition ont été notés dans 12%. La prise de can-nabis a été notée dans 12% des cas. Des antécédents familiaux de premier degré d'AVCI ont été retrouvés dans 25% des cas. Tous nos patients ont bénéficié d'une imagerie cérébrale, représentée par une an-gioIRM dans 80% et une TDM cérébrale dans 20% des cas. Le territoire sylvien était touché dans 80% des cas, suivi du territoire vértébro-basilaire (12%) et du territoire de l'artère cérébrale postérieure (8%). Le délai entre l'installation des symptômes et la première imagerie cérébrale dépassait les 12h dans 100% des cas. L'échodoppler vasculaire avait mis en évidence quelques plaques athéromateuses sans sténose significative au niveau carotidien ainsi que des épaississements pariétaux chez 12% des cas. L'échographie transoesophagienne a permis de mettre en évidence un anévrysme du septum interauriculaire chez un de nos patients, elle restait sans particularités dans le reste des cas. Le bilan autoimmun montrait la positivité des anticorps antinucléaires dans 72% des cas, les anticorps antiphopspholipides étaient positifs dans 36%, dans les deux tiers des cas un anticorps anticardiolipine a été retrouvé. Les dosages des protéines S, C et de l'antithrombine III étaient normaux. L'enquête étiologique entreprise chez ces patients a pu découler sur un diagnostic dans 76% des cas. Les étiologies étaient représentées par le lupus systémique aigu (32%) associé à un syndrome des antiphospholipides dans 80% des cas. Il faut noter que la moitié des patients lupiques avaient en plus une atteinte rénale compliquée dans les deux tiers des cas d'hypertension artérielle. Tous ces patients étaient en poussée de leur maladie avec un SLEDAI moyen 34 ± 12. Le syndrome des antiphosphilipides primitif a été retrouvé dans un cas (n = 1) qui était auparavant sous aspirine et hydroxy-chloroquine et avait un antécédent d'atteinte de l'artère centrale de la rétine. La maladie de Behcet était retrouvée dans 16%, les trois quart avaient une poussée neurologique au moment du diagnostic, la moitié avait une atteinte oculaire active au moment de l'AVCI avec un traitement immunosuppresseur en cours. La maladie de Takayasu représentait 12% des cas, deux malades présentaient une atteinte type V, et une malade présentaient une atteinte type IIA, qui était également hypertendue et avait en plus une atteinte oculaire. Une thrombophilie à type de mutation du gène de la prothrombine (G20210A) (n = 1). Une polyarthrite rhumatoïde maligne avec vascularite cérébrale (n = 1). Un AVCI sur anévrysme du septum interauriculaire sans Foramen ovale per-méable chez un patient où une maladie de Behcet restait suspectée.

Les patients ont été classés selon la classification TOAST. Ainsi, un seul patient était classé dans le TOAST II ayant présenté un anévrysme du septum interauriculaire. Les patients du TOAST III étaient à 73% fumeurs, 8% avaient en plus un diabète type II et/ou une hypertension artérielle et 12% prenaient au moment du diagnostic une con-traception oestro-progestative. Les patients répondant aux critères du TOAST IV re-présentaient 72% du total des patients. Chez 24% des patients, aucun diagnostic étiolo-gique n'a été retenu (TOAST V). L'AVCI avait révélé la maladie dans 25%. Dans les autres cas, le délai moyen entre la maladie et l'accident vasculaire était estimé à 3ans. Les patients avec un SAPL primtif ou associé ont reçu un traitement anticoagulant après la phase aigüe. Un traitement à base de corticoïdes a été entrepris dans 76% des cas, par voie orale à pleine dose dans les deux tiers des cas et par bolus de méthylpré-dnisolone dans le tiers restant. Le recours à des immunosuppresseurs à type cyclo-phosphamide de façon mensuel ou azathioprine a été nécessaire dans 76%. L'utilisation des corticoides et des immunosuppresseurs concernait essentiellement les patients lupiques ou ayant un Behcet. Le méthotrexate a été utilisé également dans deux cas présentant une maladie de Takayasu. Tous nos patients ont bénéficié de séances de rééducation physique. La prévention secondaire était faite par un antiagrégant plaquettaire dans tous les cas sauf chez nos patients qui étaient sous anticoagula-tion. L'évolution était marquée par une récupération incomplète avec séquelles motrices dans 40%. La survenue d'une dépression était notée chez 16% des patients avec un délai moyen de 13 mois post AVC, trois de ces patients avaient une maladie de Behcet et une patiente était lupique avec un SAPL associé. Des pertes de mémoires ont été no-tées dans 20% des cas, tous étaient de sexe féminin, ayant un passé migraineux et lu-pique avec un SAPL associé.

## Discussion

Les AVCI survenant chez les sujets jeunes ne sont plus une entité rare, bien au con-traire ils sont en nette augmentation ces dernières années. Les particularités de cette population sont, premièrement la fréquence d'autres étiologies en dehors des causes classiques d'AVCI qu'ils partagent avec les sujets plus âgés qui nécessitent une prise en charge spécifique, et secundo le poids des séquelles fonctionnelles, vu que ces jeunes auront des capacités réduites de façon précoce, dans une période de leur vie où ils sont censés être les plus actifs et les plus producteurs. Dans un service universitaire de médecine interne au recrutement tertiaire et spéciali-sé en maladies de système, les étiologies chez nos patients avaient un profil bien parti-culier dominé par les maladies auto-immunes et auto-inflammatoires. Il a été démontré dans de précédentes études que le risque d'hospitalisation pour un AVCI était augmen-té pour de nombreuses maladies immuno-médiées notamment le lupus érythémateux systémique et la polyarthrite rhumatoïde, il l'est d'autant plus élevé dans la première année d'hospitalisation pour de nombreuses maladies auto-immunes [6]. Dans notre série, le lupus érythémateux systémique et le SAPL soit associé ou primitif prédominaient parmi les causes retrouvées d'AVCI. La prévalence des AVCI chez les patients lupiques allait dans la littérature de 2% à 15%. Les événements cérébro-vasculaires comptent pour 20 à 30% des causes de mortalité chez les patients lupiques, ces mêmes patients avaient 2.04 fois plus de risque d'être hospitalisés pour un AVCI que la population générale [7]. En plus des facteurs classiques d'athérosclérose, les patients lupiques ont des facteurs de risque particuliers prédisposant aux événements vasculaires, ainsi une large étude espagnole a montré que la présence d'une sérite, d'un complément bas, d'une atteinte neuropsychiatrique du lupus, d'une atteinte valvu-laire ainsi que la présence d'anticorps antiphospholipides augmentaient le risque d'avoir un événement cardiovasculaire, sans avoir trouvé une association avec le score SLEDAI [8]. Pourtant une relation semble exister entre l'activité du lupus et le risque augmenté d'un événement vasculaire [9,10], dans notre étude la majorité de nos patients étaient en poussée de leur maladie avec un SLEDAI élevé au moment de l'AVCI. Un des facteurs de risque vasculaire spécifique au lupus reste l'atteinte rénale, ainsi l'existence d'une protéinurie ou l'augmentation de la créatinine sont des causes recon-nues d'athérosclérose accélérée [11] et prédisposent aux événements cardiovascu-laires en général et à l'AVCI en particulier. La moitié de nos patients lupiques avaient une atteinte rénale au décours de laquelle ils ont présenté leur AVCI et étaient traités par des immunosupresseurs.

L'existence d'anticorps antiphospholipides soit dans un SAPL primitif ou associé à d'autres maladies notamment le lupus, reste un élément prothrombotique très impor-tant. Dans l'étude de Cervera et al, où ils ont suivi 1000 patients avec un SAPL pendant 10 ans, ils ont trouvé que l'événement thrombotique le plus fréquent lors de l'évolution est l'AVCI avec une prévalence de 5,3% du total de la cohorte sans avoir trouvé des différences entre le SAPL primitif ou associé au lupus. Concernant la morta-lité, les causes les plus communes étaient les causes thrombotiques dont l'AVCI et représentaient 36,8% de toutes les causes confondues [12]. D'autres études ont analysé le risque cardiovasculaire en fonction du profil des anticorps antiphospholipides positifs, ainsi une étude suédoise a montré que la présence des anticorps anticardiolipines étaient plus liée au risque de l'AVCI qu'au risque de faire un infarctus du myocarde (10). Malgré le petit nombre de nos malades, nous avons constaté également que la présence des anticorps anticardiolipines prédominait chez nos patients. Les résultats restent controversés pour les anticorps anti B2-glycoprotéine et l'anticoagulant lupique, qui étaient plus liés aux autres événements vasculaires en dehors de l'AVCI [10-13]. La valvulopathie de Libman Sacks doit être recherchée systématiquement par échographie cardiaque transoesophagienne devant un AVCI chez un patient lupique ou ayant un SAPL. Cependant aucun patient de notre série n'avait une valvulopathie de Libman Sacks, mais il faut noter que la majorité de nos malades n'avaient eu qu'une échographie cardiaque transthoracique ce qui reste une limite dans l'exploration dia-gnostique complète d'une éventuelle valvulopathie de Libman Sacks. Avec les anticorps antiphospholipides, la présence d'une valvulopathie de Libman Sacks représente le deuxième facteur de risque le plus important d'AVCI en plus des FDR traditionnels chez les patients lupique et/ou ayant un SAPL [14]. Chaque patient ayant un Libman Sacks avec des végétations a 3 fois plus de risque d'avoir des micro-emboles cérébrales par heure et ainsi plus d'AVCI constitué ou transitoire [15]. La survenue d'un AVCI lors d'une vascularite primitive chez le jeune reste rare sauf en cas de maladie de Takayasu [16] où elle est habituelle notamment comme mode de révélation de la maladie. Dans notres série, elle représentait la troisième cause d'AVCI, la majorité de nos patients n'avaient pas d'autres facteurs cardiovasculaires à part la vascularite et avaient des lésions étendues et une maladie active. Dans le Takayasu, les études manquent dans la littérature pour évaluer isolément les AVCI, les différentes études entreprises évaluent le risque cardiovasculaire général, pourtant cette atteinte n'est pas rare et mérité d'être étudiée à part entière. Dans une étude coréenne [17], les accidents vasculaires ischémiques étaient notés dans 17.6% et représentaient fréquemment le motif initial de consultation, précédant le diagnostic du Takayasu chez tous les patients de cette étude, la majorité des patients étaient classés type V. Les co-morbidités telles que le diabète, les antécédents cérébrovasculaires ou l'hypertension artérielle ne différaient pas statistiquement entre les groupes où le Takayasu restait en activité ou pas. Concernant les mécanismes de l'AVCI dans le Takayasu, ils reposaient sur la présence d'emboles provenant d'une lésion sténotique ou occlusive au niveau de l'aorte ou ses branches, sur un terrain d'hypertension artérielle, de cardioembolisme ou dans un contexte de flux cérébral réduit [18].

Ce qui est particulier dans cette série, tient au fait que 16% des AVCI chez nos jeunes étaient dues à une maladie de Behcet. Cette maladie représentait alors la deuxième cause d'AVCI dans cette étude. Cela est dû probablement à la grande prévalence de la maladie de Behcet au Maroc et au fait que l'étude a été menée dans un centre de réfé-rence de cette maladie. Cette atteinte était associée à d'autres lésions neurologiques et à l'atteinte oculaire, et était survenue chez des patients qui étaient déjà sous traitement immunosuppresseurs et n'avaient pas de haut risque cardiovasculaire. L'atteinte arté-rielle reste très rare dans la maladie de Behcet et c'est encore plus vrai quand il s'agit d'accident ischémique cérébral, peu de cas sont rapportés jusque-là [19], dans une sé-rie marocaine cinq cas ont été rapportés [20]. Un cas de polyarthrite rhumatoïde compliqué d'AVCI a été retrouvé dans notre série. Le risque d'avoir un AVCI s'élève à 30% par rapport à la population générale chez les patients avec polyarthrite rhumatoïde selon une étude danoise, et le risque de mortali-té suite à un AVCI est estimé à 50% de plus que chez les patients sans polyarthrite rhumatoïde selon une méta-analyse [14]. Un seul cas de thrombophilie à type de mutation du gène de la prothrombine (G20210A) a été noté dans notre série. La recherche des mutations de gènes codant pour des facteurs procoagulants ou proathérosclérotiques ne fait pas partie de notre bilan de routine et n'est faite que dans les cas d'AVCI chez les jeunes dont l'étiologie reste obscure malgré un bilan exhaustif, et c'est également ce qui est recommandé dans la littérature [16,21]. L'existence d'un polymoprhisme génétique est actuellement considérée comme un FDR indépendant, qui augmente le risque global d'avoir un AVCI, notamment chez les jeunes [21]. Il faut noter également la forte prévalence du cannabisme dans cette série, mais aucune recherche toxicologique n'a été faite au moment du diagnostic et le délai entre la der-nière consommation et l'accident vasculaire n'a pas été précisé dans les dossiers médicaux. Une cause de plus en plus retrouvée à l'origine d'ischémie cérébrale chez les jeunes, qu'il faut rechercher à l'anamnèse, car c'est une cause également de récur-rence. Il est nécessaire d'accompagner le patient dans un processus de sevrage, cela concerne également les autres toxiques (opiodes, cocaine…). Plusieurs mécanismes ont été avancés pour expliquer le lien entre le cannabis et l'AVCI comme un vasospasme réversible, une hypoperfusion cérébrale par effet sympathique périphérique, et surtout un vascularite cérébrale notamment après la description de cas de thromboangéite des membres inférieurs rappelant une maladie de Buerger chez certains patients [22].

Les dissections artérielles et les emboles d'origine cardiaque restent les causes les plus fréquentes d'AVCI chez les jeunes dans la littérature [16]. Au Maroc, la cardiopa-thie emboligène constitue la première cause dans notre pays, vu que la valvulopathie rhumatismale est encore bien présente dans notre contexte. L'absence de ces deux étiologies dans notre série est expliqué par le mode de sélection vu le recrutement es-sentiel de maladies de système au niveau du service. Un seul cas a été suspecté d'embolisme d'origine cardiaque vu la présence d'un anévrysme du septum interventriculaire mais qui reste une cause exceptionnelle d'AVCI en l'absence d'un foramen oval persistant, la présence d'aphtose buccale à répétition fait suspecter chez lui une maladie de Behcet. Vu que la majorité de nos malades avaient des maladies systémiques avec une base autoimmune ou autoinflammatoire le traitement étiologique était constitué essentielle-ment de corticoides et d'imunosuppresseurs. Le bénéfice lié aux effets anti-inflammatoires et immunomodulateurs de la corticothérapie pourrait, par un meilleur contrôle de l'activité de la maladie, être supérieur à l'effet pro-athérogène induit par le traitement [23].

## Conclusion

Cette étude a comme point faible essentiel le petit nombre des patients inclus, car elle a été faite dans un service de médecine interne non spécialisé en pathologie vasculaire cérébrale avec de nombreux biais de sélection. Mais elle a permis de jeter de la lumière sur des étiologies rares d'AVCI qui pourraient survenir chez les jeunes, dont la connaissance pourrait améliorer le pronostic, réduire les séquelles et éviter la récurrence qui pourrait être fatale, en proposant un traitement étiologique adéquat.

### Etat des connaissances actuelle sur le sujet

- Les ischémies cérébrales chez le sujet jeune sont de plus en plus fréquentes;
- Les étiologies des accidents cérébrales chez les jeunes sont différentes.

### Contribution de notre étude à la connaissance

- Elle met en exergue les étiologies particulières qui pourraient être responsables d'ischémie cérébrale chez les jeunes, afin d'attirer l'attention des praticiens vers ces causes;
- Après le bilan étiologique, cette étude apporte également une idée sur les traitements à visée étiologique dont pourraient bénéficier les patients jeunes en plus du traitement symptomatique;
- Elle montre également le rôle que pourrait jouer l'interniste dans la prise en charge de cette pathologie à côté du neurologue notamment dans le volet étiologique.

## Conflits d’intérêts

Les auteurs ne déclarent aucun conflit d'intérêts.
